# Surgical treatment for lung metastasis of inflammatory myofibroblastic tumor of the lung: A case report

**DOI:** 10.1111/1759-7714.14914

**Published:** 2023-05-05

**Authors:** Hidenobu Iwai, Naoki Yanagawa, Hiroyuki Deguchi, Makoto Tomoyasu, Wataru Shigeeda, Yuka Kaneko, Ryuichi Yoshimura, Hironaga Kanno, Mayu Sugai, Shunsuke Shikanai, Tamotsu Sugai, Hajime Saito

**Affiliations:** ^1^ Department of Thoracic Surgery Iwate Medical University Iwate Japan; ^2^ Department of Molecular Diagnostic Pathology Iwate Medical University Iwate Japan

**Keywords:** inflammatory myofibroblastic tumor, lung metastasis, lung tumor, recurrence, surgery

## Abstract

Inflammatory myofibroblastic tumor (IMT) is a rare disease that is considered an intermediate neoplasm, with the risk of recurrence and metastasis. Surgical treatment is the standard therapy for IMT, although there are only a few reports of surgery for lung metastasis of pulmonary IMT. We opine that surgical treatment might be effective not only for localized tumors, but also for cases of lung metastasis of IMT.

## Graphical abstract

A left lower lobectomy with hilar and mediastinal lymph node dissection was performed for a cord‐like shadow in the left lower lung lobe, and histopathologic and immunohistochemical examination revealed a diagnosis of inflammatory myofibroblastic tumor (IMT). Nine months after surgery, a nodule was found in the right middle lobe, which rapidly expanded over the next three months, so a right middle lobe wedge resection was performed, and the resected lesion was diagnosed as a pulmonary metastasis of IMT. There has been no recurrence, and we propose to consider surgical resection as a treatment option for pulmonary metastasis of IMT.

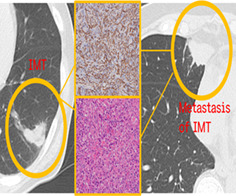

## INTRODUCTION

Inflammatory myofibroblastic tumor (IMT) is a rare disease, accounting for about 0.04–0.18% of all lung lesions.^1^, ^2^, ^3^ IMT arise from various organs and are often treated as malignant rather than benign tumors because of their potential to metastasize and recur. Surgical treatment is the standard therapy for IMT, although radiation and chemotherapy have also been tried in a small number of cases.^4^ The preferred treatment for unresectable or recurrent cases, on the other hand, is unknown. Here, we report a case of lung metastases of IMT that was successfully surgically treated.

## CASE REPORT

**FIGURE 1:**
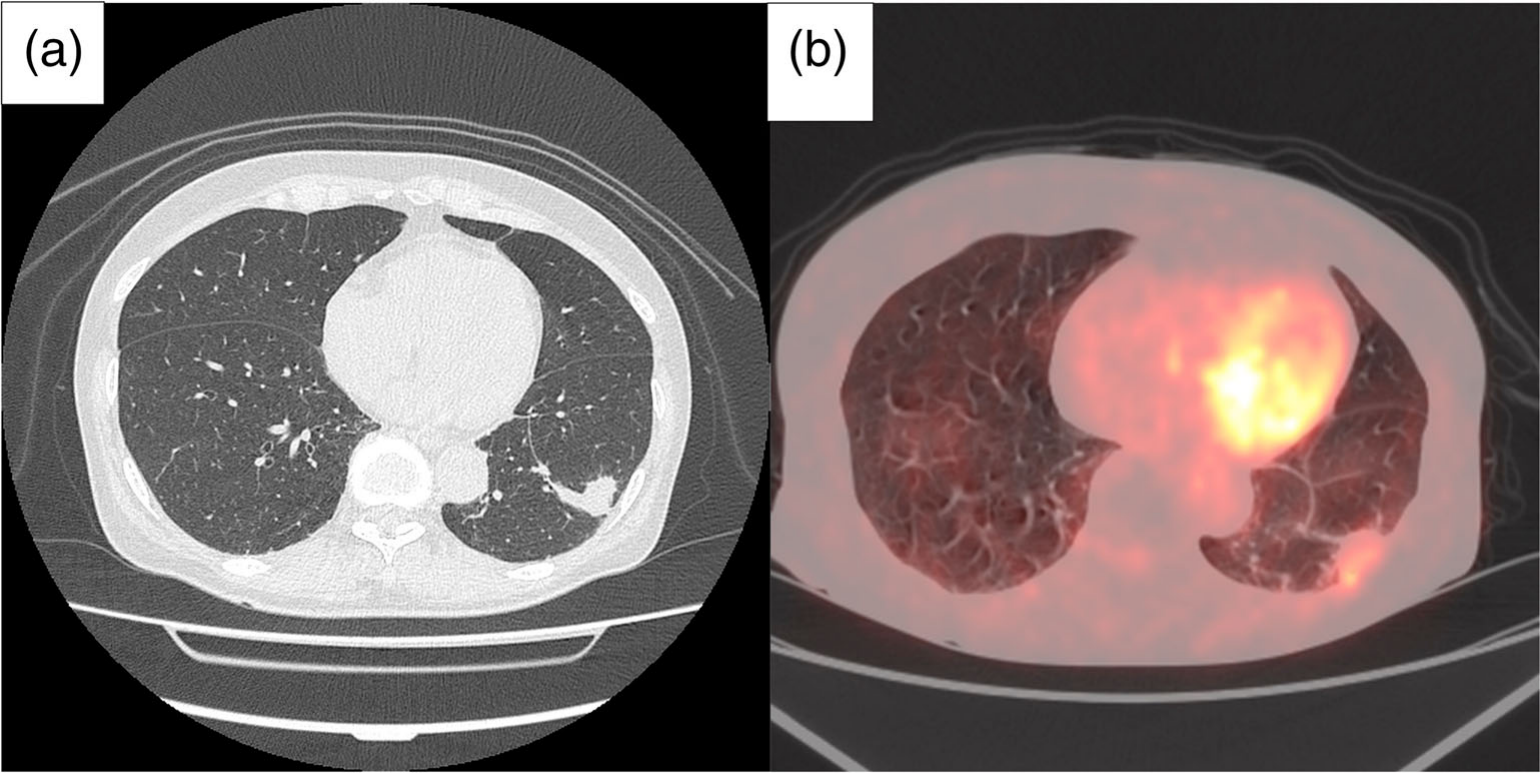
HRCT showed the appearance of a new nodule near the cord‐like shadow (a). PET/CT revealed 18F‐fluorodeoxyglucose (FDG) uptake in the mass (maximum standard uptake value = 2.8) (b).

A 69‐year‐old female was incidentally found to have a cord‐like shadow in the left lower lung lobe, which was identified on high‐resolution computed tomography (HRCT) of the chest, for which she was under observation. Three years later, HRCT showed the appearance of a new nodule near the cord‐like shadow (Figure 1a).

Bronchoscopy was performed for diagnosis of the newly emerging shadow, but the diagnosis could not be confirmed. Positron emission tomography/computed tomography (PET/CT) revealed 18F‐fluorodeoxyglucose (FDG) uptake in the mass (maximum standard uptake value = 2.8) (Figure 1b). She was referred to our department for diagnostic and therapeutic surgery, and we performed a three‐port video‐assisted thoracoscopic surgery. Rapid pathological diagnosis showed no obvious malignant findings, but the possibility of lung cancer could not be ruled out clinically and we performed a left lower lobectomy with hilar and mediastinal lymph node dissection.

**FIGURE 2:**
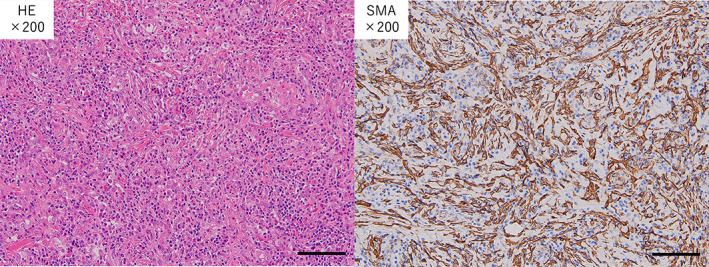
Histopathological evaluation showed no obvious atypical cells, but demonstrated proliferation of inflammatory cells, fibroblasts and collagen fibers. Bar = 100 μm (a). Immunohistochemistry showed that the tumor cells were positive for smooth muscle actin (SMA), suggesting that they were myofibroblasts. Bar = 100 μm (b). HE, Hematoxylin‐Eosin.

The tumor was 45 × 24 × 20 mm in size. Histopathological examination revealed no obvious atypical cells, but showed proliferation of inflammatory cells, fibroblasts, and collagen fibers, suggesting inflammatory changes (Figure 2a). Immunohistochemical evaluation showed that the tumor cells were positive for smooth muscle actin (SMA) (Figure 2b) and negative for anaplastic lymphoma kinase (ALK) fusion gene, c‐ros oncogene 1 (ROS1) fusion gene, desmin, and cytokeratin AE1/AE3. The percentage of Ki‐67 positive cells was 3%. There were no malignant findings in the lymph nodes. These findings led to the diagnosis of IMT.

**FIGURE 3:**
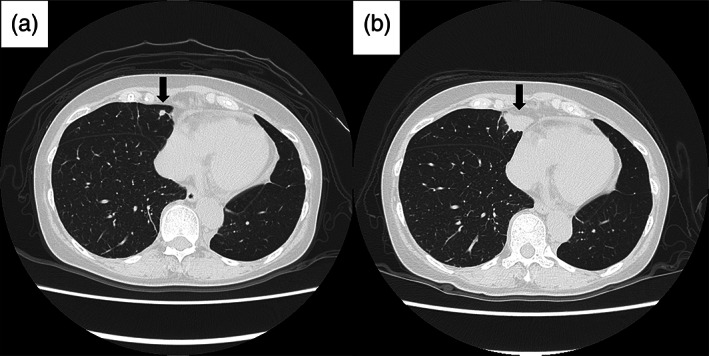
Nine months after surgery, HRCT showed the appearance of a nodule in the right middle lobe (a). One year after surgery, the right middle lobe nodule enlarged rapidly (b).

**FIGURE 4:**
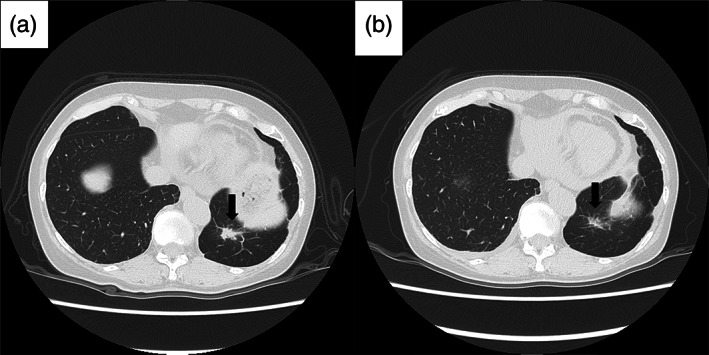
A year after surgery, a nodule with irregularly shaped margins appeared at the base of the residual left upper lobe (a). One month after the second surgery, HRCT showed disappearance of the nodule in the residual left upper lobe (b).

Nine months after surgery, HRCT showed the appearance of a nodule in the right middle lung lobe, which rapidly enlarged within 3 months, along with development of a nodule with irregularly‐shaped margins at the base of the residual left upper lung lobe (Figures 3 and 4a). As PET/CT revealed FDG uptake in the right middle lobe mass (maximum standard uptake value = 3.38), we considered the possibility of lung metastases of IMT. As the lesions were localized to the lung, we decided to perform surgical resection for both the lesions and accordingly thoracoscopic right middle lobe wedge resection was performed. Histopathological evaluation and the results of immunostaining were consistent with the findings of the left lower lobe lesion, and hence we diagnosed the resected lesion as pulmonary metastasis of IMT. HRCT performed 1 month after the second surgery showed disappearance of the nodule in the residual left upper lobe (Figure 4b) and we have been continuously following up the patient with no further recurrence for 8 months.

## DISCUSSION

In 2018, the World Health Organization classified IMT as “fibromatous neoplasms, uncertain whether benign or malignant” (ICD‐11). IMT is a neoplasm composed of myofibroblastic and fibroblastic spindle cells, accompanied by inflammatory cells. IMT has been described in nearly every organ, although the lung is one of the most common locations for IMT.^5^

Pathological diagnosis is very important for IMT, and immunostaining is generally reported to be positive for vimentin, SMA, and desmin, while cytokeratins, CD34, c‐kit, and S100 protein are reportedly negative.^3^, ^6^

IMT is usually treated surgically if complete resection of the lesion is possible. Another important objective of surgical excision is obtaining a more reliable diagnosis. Complete surgical resection has a good prognosis, with a 5‐year survival rate of 91%. Local recurrence is reported in less than 2% of cases and distant metastasis in less than 5%.^7^

Generally, when surgical treatment is not possible, other treatments might be used as an alternative. Although radiation therapy at various doses has been reported, the appropriate dose has not been established.^8^, ^9^

Chemotherapy is used for systemic lesions, and regimens with platinum have been reported to be beneficial.^10^, ^11^ There have also been reports of tyrosine kinase inhibitors (TKIs) being effective in patients who are positive for genetic mutations, such as ALK and ROS1,^12^, ^13^ and in light of the results with TKIs for lung tumors to date, screening for genetic mutations might become mandatory in IMT cases in the future. Yamamoto et al. reported that 72.5% of the patients they reviewed were positive for ALK mutations and 5% were positive for ROS1.^14^ Based on these reports, we performed gene panel testing using next‐generation sequencing to confirm the presence of genetic mutations in this case, although the test was not successful because a tumor fraction of more than 30% was not obtained. It should be noted that the tumor in our case was composed mainly of inflammatory cells, which might have prevented gene mutation testing due to the lack of sufficient tumor cells, which is a lesson for the future.

Our patient has since progressed without local recurrence, suggesting that surgical treatment is an effective option for metastatic lesions that can be completely resected.

## CONFLICT OF INTEREST STATEMENT

The authors declare that there are no conflicts of interest. This research received no specific grant from any funding agency in the public, commercial, or not‐for‐profit sectors.
